# Human peri‐gastruloids: a significant advancement in embryology research

**DOI:** 10.1002/mco2.445

**Published:** 2023-12-30

**Authors:** Qin Yang, Ezra Burstein, Da Jia

**Affiliations:** ^1^ Key Laboratory of Birth Defects and Related Diseases of Women and Children Department of Paediatrics West China Second University Hospital, State Key Laboratory of Biotherapy Sichuan University Chengdu China; ^2^ Department of Internal Medicine University of Texas Southwestern Medical Center Dallas Texas USA

## Abstract

The peri‐gastruloids comprise both embryonic (epiblast) and extraembryonic (hypoblast) tissues, faithfully mirroring crucial developmental events spanning from the immediate post‐implantation phase to early organogenesis, encompassing the emergence of amniotic and yolk sac cavities, as well as the progression from bilaminar to trilaminar embryonic discs.

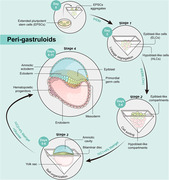

1

In a recent study published in *Cell*, Liu L et al.^1^ reported a strong and effective technique that facilitates human extended pluripotent stem cells (hEPSCs) to spontaneously arrange into structures resembling embryos, referred peri‐gastruloids. These peri‐gastruloids comprise both embryonic (epiblast) and extraembryonic (hypoblast) tissues, faithfully mirroring crucial developmental events spanning from the immediate post‐implantation phase to early organogenesis. The main advance of peri‐gastruloids over previously human gastruloids is the abundance of hypoblast produced, providing essential signals for patterning the developing foetus. Through single‐cell RNA‐sequencing, the researchers revealed that these structures give rise to cell lineages with remarkable transcriptomic similarities between peri‐gastruloids and primate embryos at the single‐cell level. This innovative peri‐gastruloid platform opens up fascinating possibilities for further exploration beyond the gastrulation phase and holds great potential for harnessing physiologic developmental processes towards human tissue engineering, potentially leading to applications in regenerative medicine.

Developing mammalian embryos self‐organize to oversee complex morphogenetic processes, ultimately resulting in the formation of a fully developed organism. Gastrulation is a complex process during early embryonic development, characterized by the differentiation into three primary germ layers (endoderm, mesoderm, and ectoderm) and the establishment of the fundamental body plan, which imparts spatial polarity across all three dimensions. This intricate transformation marks a pivotal step in shaping the embryo's future structure and function. However, due to bioethical constraints, the cultivation of human embryos is strictly limited to 14 days, preventing the study of gastrulation, which occurs between approximately 14 and 21 days post‐fertilization. Consequently, gastrulation is considered the “black box” of human development. In order to understand this back box, various early embryo models have been constructed based on either human embryonic stem cells or human‐induced pluripotent stem cells.^2^ Unfortunately, the models lack key extraembryonic tissues such as the yolk sac (YS) and cannot faithfully mimic natural embryo development process. Hence, the establishment of an integrated model that faithfully replicates critical phenomena occurring in human peri‐gastrulation development remains an ongoing challenge.

The peri‐gastruloid models, developed by Liu et al.,^1^ enable human‐extended pluripotent stem cells (hEPSCs) to form structures resembling embryos. Peri‐gastruloids effectively recapitulate human post‐implantation development through the early stages of organogenesis, encompassing the emergence of amniotic and YS cavities, as well as the progression from bilaminar to trilaminar embryonic discs. The peri‐gastruloid models are nonviable due to the absence of trophoblast cell that form the placenta, thus helping to clear ethical concerns in this research. The authors observed various embryonic structures and tissues in peri‐gastruloids, such as the primitive streak (PS), neural plate, neural tube, tail bud, somites, heart tube, gut tube, and limb buds. By performing single‐cell RNA sequencing, the authors discerned 13 predominant cell clusters that correspond to significant cell types in the context of human development during the peri‐gastrulation stage. The analytical findings unveiled the existence of hematopoietic progenitors, indicating the initiation of the initial hematopoietic wave within YS‐like structures. They also compared the transcriptomic profiles of peri‐gastruloids with those of natural primate embryos and found high similarities.

The peri‐gastruloids are formed in four stages (Figure 1). In the first phase of culture, the authors used a titrated hypoblast differentiation medium to induce hEPSCs to differentiate into epiblast‐like cells and hypoblast‐like cells. In the second stage, peri‐gastruloids consisting of two main compartments, both the epiblast‐like compartment and the hypoblast‐like compartment underwent significant growth on days 3–4. In the third phase of culture, bilaminar‐disc‐like structures appeared on days 5–6. Notably, the addition of Matrigel improved the development of the amniotic‐like cavity. Finally, discernible trilaminar‐disc‐like structures were evident on day 8. These structures replicate the disruption of symmetry and the establishment of the anterior–posterior axis within the epiblast‐like compartment, providing a model for elucidating PS‐like structures and the gastrulation process in humans. Following the formation of these structures, signs of early neurulation and organogenesis become apparent.

**FIGURE 1:**
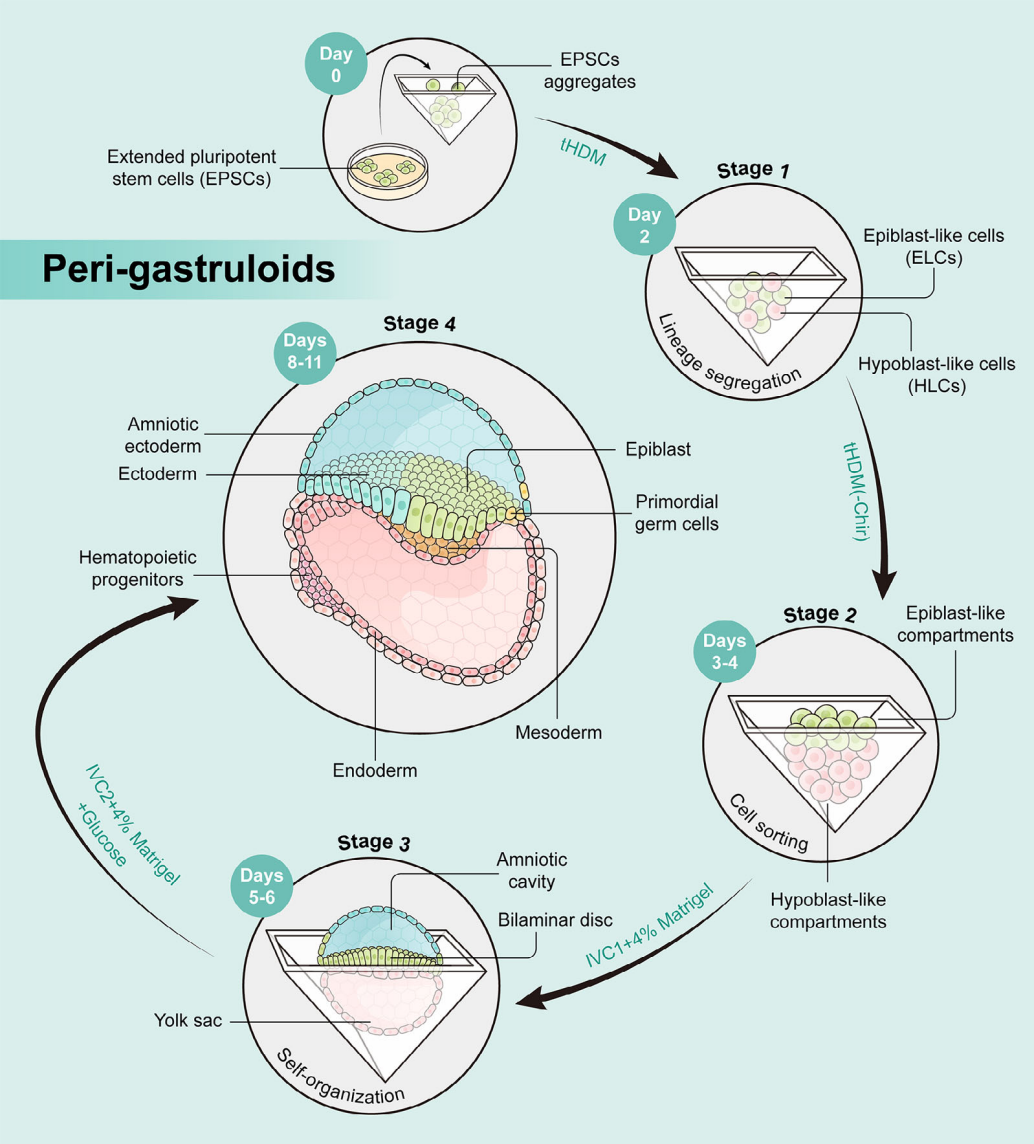
Schematic illustrating the critical stages in the formation of peri‐gastruloids, modified from Liu et al.^1^ The formation of peri‐gastruloid mainly consists of four key stages. Stage 1: human‐extended pluripotent stem cells (hEPSCs) are induced to differentiate into epiblast‐like cells (ELCs) and hypoblast‐like cells (HLCs). Stage 2: the formation of epiblast‐like and hypoblast‐like compartments. Stage 3: the development of amniotic‐like and primary yolk‐sac‐like cavities, accompanied by the appearance of bilaminar‐disc‐like structures. Stage 4: the formation of primitive‐streak‐like and trilaminar‐disc‐like structures and the commencement of early organogenesis.

To summarize, the peri‐gastruloid model represents a great breakthrough in the field of embryology for several reasons. First, compared to recently reported post‐implantation embryos,^3^, ^4^ the peri‐gastruloid model utilizes a relatively simple experimental methodology, necessitating neither transgene‐directed differentiation nor complex manipulation. It begins with a single‐cell type and involves minimal intervention. Second, peri‐gastruloids demonstrate appropriate lineage allocation and signaling activities, as well as morphological features that resemble natural primate embryos at different developmental stages. Last, the peri‐gastruloids offer an invaluable avenue for exploring both human development and pathological conditions. Moreover, comparative studies between this human model and similar models in other mammals may help unlock early developmental mechanisms that determine species‐specific characteristics, including body size, complexity of the nervous system, and many other features that define the human body. In the meantime, the model can also serve as a robust platform for evaluating pharmaceutical agents and their potential embryologic toxicities, as well as the potential of therapeutic interventions in early embryogenesis. By comprehending the molecular and cellular mechanisms of embryonic development, it will provide a theoretical foundation for advancing clinical practices in the diagnosis, prevention, and treatment of congenital genetic defects.

Despite the significant potential of the peri‐gastruloid model, its limitations should also be noted. First, peri‐gastruloids inadequately replicate the intricate nature of the human embryo, lacking trophoblast tissues and a chorionic cavity. This deficiency impedes its capacity to faithfully replicate the intricate developmental processes of a human embryo. Second, the developmental progression of peri‐gastruloids is constrained, typically limited to 11–13 days. To enable the ongoing research of peri‐gastruloids, further modifications will be necessary. For instance, the authors speculate that incorporating human cord blood serum and employing a roller culture system with precise control over oxygenation and pressure, as demonstrated in previous mouse studies,^5^ could allow the model to progress past its current developmental stage. Last but not the least, ethical issues abound. The peri‐gastruloid model offers a unique opportunity to unlock the mysteries of early human development, but as the advancements in these models continue to push the developmental stages achieved, the development of a functional fetus in an in vitro system is becoming a potential outcome of these technologies. Therefore, it is time to initiate a discussion about regulating the extent of in vitro development, in order to achieve equilibrium between research opportunities and rationalities. Ensuring that the emerging models' features align with the potential societal benefits is of utmost importance.

## AUTHOR CONTRIBUTIONS

Q.Y., E.B., and D.J. wrote the paper. All authors have read and approved the article.

## CONFLICT OF INTEREST STATEMENT

The authors declare they have no conflicts of interest.

## ETHICS STATEMENT

Not applicable.

## Data Availability

Not applicable.
